# Correlation of Tumor Budding With Known Clinicopathological, Histomorphological and Hormonal Receptor Status in Patients With Invasive Breast Carcinoma

**DOI:** 10.7759/cureus.29637

**Published:** 2022-09-26

**Authors:** Gunvanti B Rathod, Killol N Desai, Atul Shrivastava, Alpeshkumar M Maru

**Affiliations:** 1 Pathology, AIl India Institute of Medical Sciences (AIIMS), Hyderabad, IND; 2 Pathology, Nootan Medical College & Research Centre, Visnagar, IND; 3 Pathology, Gujarat Medical & Education Research Society (GMERS) Medical College and Civil Hospital, Gandhinagar, IND; 4 Pathology, Dr. N. D. Desai Faculty of Medical Science & Research Centre, Nadiad, IND

**Keywords:** histomorphology, breast carcinoma, immunohistochemistry, infiltrating ductal carcinoma, tumor buds

## Abstract

*Introduction*: Tumor blossoming may be a predictive indicator for a variety of cancers. At the invasive origin of the tumor, cells get detached from the original tumor mass.

*Aims & objectives*: Studying breast cancer tumor budding, as well as its link to other prognostic indicators, such as clinicopathological features and hormone receptor status, will be the focus of this study.

*Materials & methods*: Over six years, 110 cases of invasive breast cancer were examined. Ten high-power fields were used to analyze H&E-stained slices for tumor sprouting. It was determined that the tumor buds were divided into low and high grades. Tumor budding and other prognostic factors were compared using the chi-square test. It was considered significant if the p-value was less than or equal to 0.05.

*Results*: There were 110 cases of invasive ductal carcinoma, which accounts for more than half of the total cases (88.18%). A total of 144 tumors were present, of which 74 displayed strong budding and 36 displayed poor budding. A correlation between tumor budding and tumor size, lymph node metastasis, and tumor stage is statistically significant (P = 0.0099).

*Conclusion*: Tumor budding in breast cancer is an easily visible in microscopy, novel prognostic indicator. A new prognostic element may be added to the reporting process.

## Introduction

Carcinoma of the breast is the second largest cause of mortality from cancer among women in India [1]. Breast lumps and other constitutional symptoms were the most common clinical presentations [2]. Cancer patients' prognosis is affected by a number of variables, including age, tumor kind, grade and stage, and the presence or absence of a hormone receptor. It is the goal of all of these methods to ensure that the proper therapy is given to the right patients [3]. Improved breast cancer detection and treatment have contributed to a drop in mortality over the last several decades. Biomarkers and other prognostic criteria need to be given much more consideration.

Tumor budding is one of these prognostic indicators. Detachment from neoplastic glands at the invasive front of the tumor means a limited number of cancer cells that are separated from the main tumor mass [4]. Peri tumoral buds and intra-tumoral buds are the terms used to describe tumor buds that are positioned on the outside of a tumor mass and those that are located inside the tumor mass [2].

Tumor budding has been highly recommended as a crucial step in the treatment of invasive breast cancer [5]. Various additional malignancies, such as colorectal carcinoma, gastric adenocarcinoma, and esophageal squamous cell carcinoma, have been suggested to have tumor budding as a potential prognostic marker [6, 7]. As has been demonstrated in several studies, colorectal cancer tumor buds have a role in stage II. Patients with tumor buds had a worse overall survival rate than those who did not have tumor buds [6].

EMT (epithelial-mesenchymal transition) or plasticity is the most important phase in the progression of any tumor to metastasis [7]. Cells in tumor buds may be in a partial stage of EMT, in which their connections among cells are retained, cells stay attached inside buds, and cells travel together along with circulation to the metastatic location [3].

Tumor budding in infiltrating breast cancer was studied to see whether it had any clinical or pathological importance, as well as to see if it was associated with the presence or absence of hormone receptors.

## Materials and methods

This study was conducted in the department of histopathology over six years, from December 2014 to November 2020. This study was a prospective observational type of study. Ethical approval was taken from the institutional ethical committee of Dr. N. D. Desai Faculty of medical sciences, Dharmsinh University, with IRB number NDDFMSR/IEC/24/2020. Specimens from modified radical mastectomy were included in the study, and core biopsy specimens were excluded, patients who had undergone chemotherapy were excluded.

Few patients came from outlying hospitals; most were recommended by the surgical department. The samples were fixed with 10% Neutral Buffered Formalin (NBF), and grossing was performed. In addition to the sections from the tumor and the nipple-areola complex, at least three more representative sections were obtained from all four quadrants of the breast. Hematoxylin & Eosin staining was used to examine the morphology of the slides.

To grade breast tumors, we used the Nottingham modified Bloom Richardson method and followed the WHO categorization of breast cancer [8].

Tubule development, nuclear grade, and mitosis are all included in this Bloom-Richardson (BR) grading. A poly-l-lysine coated slide was dewaxed, antigen retrieval was performed, and the sp1clone rabbit monoclonal antibody against ER (estrogen receptor), Her2 (human epidermal growth factor receptor 2), PR (progesterone receptor) antigens were then applied. The Diaminobenzidine (DAB) chromogen counterstain was used in the last phase of HRP polymerization. The Allred score [9,10] was used to record ER/PR and Immunohistochemistry (IHC) markers, whereas the American Society of Clinical Oncology's criteria of 2016 for HER2/neu reporting were used [11].

Analysis of tumor buds

Under the scanner, an invasive front of breast carcinoma was observed, and tumor buds were searched at low power. Further details were taken under the high-power field. Using a microscope with a magnification of 10x, we counted the number of tumor buds in each instance before categorizing them. When it comes to density, the two types of tumor buds are categorized as low- and high-grade tumor buds, respectively.

Analyses were conducted using Epi-info software, which measures frequency distribution. The chi-square test was used to examine tumor budding, clinicopathological characteristics, and hormone receptor status. It was determined that a P-value of 0.05 was considered statistically significant.

## Results

One hundred and ten instances of invasive breast cancer were examined in this research. Most of the patients were between the ages of 51 and 60. The first table is the age-wise distribution of the cases included (Table 1).

**Table 1:** Age-wise distribution of cases of breast carcinoma

Age group (Years)	Cases
20-30	01
31-40	03
41-50	30
51-60	56
>60	20

In the study of 110 cases of ductal carcinoma (DC), the most common kind was infiltrating ductal carcinoma (IDC), which accounted for 88.18% of the cases. The other types were invasive lobular carcinoma, invasive papillary carcinoma, and metaplastic carcinoma (1.81 percent) (Table 2).

**Table 2:** Tumor budding in various cases of breast carcinoma

Diagnosis	Cases	High Tumor budding (>20/10 HPF) = 74 cases	Low Tumor budding (≤20/10 HPF) = 36 cases
Infiltrating ductal carcinoma NOS	97	66	31
Invasive Lobular carcinoma	06	06	00
Invasive mucinous carcinoma	02	00	02
Invasive papillary carcinoma	03	01	02
Metaplastic carcinoma	02	01	01

**Figure 1:**
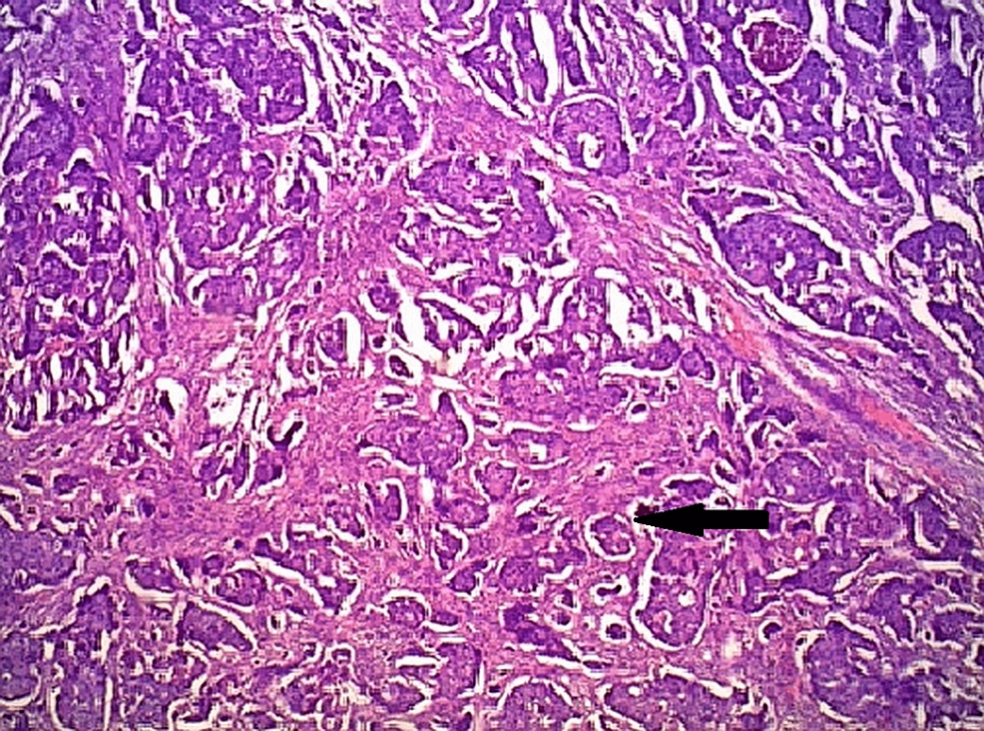
Infiltrating ductal carcinoma (H & E section) H & E: Hematoxylin and eosin section

Invasive breast carcinoma more commonly involved the left breast (60 cases) than the right breast (50 cases), and the most common site was the outer upper quadrant of the breast. Tumor budding was examined in each of the 110 cases. Tumor budding was further classified into high tumor budding (tumor buds > 20/10 HPF) and low tumor budding. (Tumor Buds 20/10 HPF) The photomicrographs of tumor budding are shown in Figure 1.

In 74 instances, tumor budding was found to be high, whereas, in 36 instances, tumor budding was found to be low. The Infiltrating ductal carcinoma NOS (IDC NOS) type (89.18 percent) and the invasive lobular carcinoma type (8.10 percent) had the highest rates of tumor budding, whereas the rates of tumor budding in mucinous carcinoma and papillary carcinoma were the lowest.

Various clinical and histomorphological aspects of tumor budding were shown in Table 3.

**Table 3:** Tumor budding & association with clinical and histopathological parameters

Parameters		High Tumor budding = 74 cases	Low Tumor budding = 36 cases	P (chi square test)
Age (cut off – 50)	≤50 years	14	20	0.00009
	>50 years	60	16	
Tumor size(cm)	≤5 cm	26	22	0.00990
	>5 cm	48	14	
Nottingham score	1 & 2	14	02	0.06215
	3	60	34	
Lymph node metastasis	Present	54	12	0.00006
	Absent	20	24	
Tumor necrosis	Present	70	14	<0.05
	Absent	04	22	

Nearly 81% of the patients with high tumor budding were over 50 years old, and 64% of the patients with high tumor budding had tumors that were at least 5 cm across. Lymph node metastasis and necrosis (72.9%) were closely linked in patients with grade 3 tumor budding. There were 54 cases of lymph node metastasis and 70 cases of necrosis in the 74 individuals who had high tumor budding levels.

As shown in Tables 4, 5, tumor budding was shown to be associated with both main staging and regional lymph node staging. It reflected that cases with T3 and T4 stages had high tumor budding (77.41%), while in the case of nodal staging, pN2 & pN3 were associated with high tumor budding (93.7%).

**Table 4:** Association of tumor budding with primary tumor staging

Stage	Cases (n=110)	High tumor budding	Low tumor budding	P value
T1 & T2	48	26	22	P = 0.0099
T3 & T4	62	48	14	

**Table 5:** Association of tumor budding with regional lymph node staging

Stage	Cases (n=110)	High tumor budding	Low tumor budding	P value
pN0 & pN1	78	44	34	P = 0.00015
pN2 & pN3	32	30	02	

We performed immunohistochemistry markers (IHC) in all malignant cases. Among them, the majority of cases with ER positivity were associated with low tumor budding, and it was statistically significant (p = 0.04), while the other IHC marker HER2/neu was not found to be statistically significant with high tumor budding as most of the HER2neu negative patients were also associated with high tumor budding.

## Discussion

Tumor budding is being recognized as a useful prognostic factor [12]. It has been used in many solid organ malignancies as a prognostic marker [13]. We see a wide range of breast cancer lesions, each of which can be classified according to one of several morphological subtypes and several clinical and histological characteristics. Tumor budding and its relationship with other prognostic variables were the focus of this investigation.

Tumor budding in carcinoma breasts represents an epithelial-mesenchymal transition (EMT). Tumor budding at an invasive front is likely to be the first step for invasion and metastasis in this EMT phenotype. Tumor budding, along with lymph node metastasis, BR score (grading), and receptor status, is thus a very sensitive and important indicator of the nature of the tumor. One can use this parameter as a complement to the traditional prognostic marker in the case of carcinoma breast because tumor budding demonstrates the poorly differentiated cell component, which is associated with poor prognosis. Reproducibility and availability of prognostic variables are required by recommendations for prognostic factor research. So far, different studies have utilized different methods for the assessment of tumor budding [14]. So various factors like cut-off value, the number of fields counted, power of the objective lens, stain used for assessment of tumor buds, and range of tumor buds were different in the size, form, histological features and showed variations. It is advisable to set standardized criteria for the assessment of tumor buds which will bring uniformity in all future studies.

In the present study, the age range of cases was a majority in between 51 and 60 years, which was comparable with the study of BN Kumarguru et al. [15], Salhia B et al. [16], and Liang F et al. [17]. The majority of the studies considered only invasive breast cancer NOS. In contrast, the current study included various histological types of breast cancer. It's because tumor budding from a ruptured tumor capsule is a common pathway shared by all types of breast carcinoma [18]. 

The researchers in the BN Kumarguru et al. [15], Salhia B et al. [16], and this study counted tumor buds in 10 fields using an X40 (HPF) objective lens. In contrast, Liang F et al. [17] used an X20 (HPF) objective lens and counted tumor buds in five fields. It is advisable to count tumor buds in 10 fields for good accuracy.

BN Kumarguru et al. [15] and the present study used similar cut-off values for tumor budding. Other studies might have used different cut-offs. BN Kumarguru et al. [15] and the present study used H & E stained sections to calculate tumor buds. In contrast, Salhia B et al. [16] used IHC-stained sections, while Liang F et al. utilized both H&E and IHC for counting tumor buds.

There was a clear correlation found between high tumor budding and the size of the tumor, the number of lymph nodes that were metastasized, and the stage of cancer in every other study that looked into the topic [16]. Our results were in line with theirs, to a degree. The correlation between necrosis and increased tumor budding was found in this investigation. Gujam FJA et al. [19], on the other hand, found it to be insignificant.

High tumor budding has been observed to be linked to tumor growth in studies by Agarwal R et al. [14], Liang F [17], and the present investigation (P 0.009). Previous studies have found an association between high tumor budding and regional lymph node metastasis (P= 0.00006), while others, such as Salhia B et al. [16] (P= 0.003) and Liang F et al. [17] (P= 0.050), as well as the current study (P= 0.00006), have found an association between high tumor budding and tumor metastasis (P= 0.046). Our study is correlated well with other studies. In the present study, we found a correlation between tumor budding and ER-positive tumors (P= 0.04). Salhia B et al. [16] found similar results (P = 0.02), but other IHC receptor statuses were not linked with tumor budding in any way.

Tumor budding has previously been shown to be an independent prognostic factor in investigations of esophageal and colon cancer tumors [20] because EMT causes cells to lose junctions, basal polarity, and signaling programs. Thus, motile tumor cells are responsible for the invasive phenotype. Tumor budding is an important mechanism of invasion and metastasis.

The study limitations are that the tumor budding has no standardized evaluation criteria, and it was done on very limited sample size. 

## Conclusions

There is a strong correlation between tumor budding and poor prognostic variables such as tumor size, lymph node metastases, and the advanced stage of the disease. ER receptor status was also shown to be related to increased tumor budding in this research. However, no statistically significant association was found with other factors like age, tumor grade, PR, and HER2neu receptor status. Despite the lack of standardized criteria for tumor budding evaluation, all reported systems provided reproducible and conclusive data, allowing us to include it as a new prognostic factor in the reporting protocol. Using the results of this investigation, we may advocate tumor budding as an easy-to-identify, unique prognostic indicator for breast cancer.
